# Optimizing the Dutch newborn screening for congenital hypothyroidism by incorporating amino acids and acylcarnitines in a machine learning-based model

**DOI:** 10.1530/ETJ-23-0141

**Published:** 2023-11-03

**Authors:** Heleen I Jansen, Marije van Haeringen, Marelle J Bouva, Wendy P J den Elzen, Eveline Bruinstroop, Catharina P B van der Ploeg, A S Paul van Trotsenburg, Nitash Zwaveling-Soonawala, Annemieke C Heijboer, Annet M Bosch, Robert de Jonge, Mark Hoogendoorn, Anita Boelen

**Affiliations:** 1 Department of Laboratory Medicine, Endocrine Laboratory, Amsterdam UMC location Vrije Universiteit Amsterdam, Boelelaan, Amsterdam, The Netherlands; 2 Amsterdam Gastroenterology, Endocrinology and Metabolism, Amsterdam, The Netherlands; 3 Department of Laboratory Medicine, Endocrine Laboratory, Amsterdam UMC location University of Amsterdam, Meibergdreef, Amsterdam, The Netherlands; 4 Department of Computer Science, Vrije Universiteit, Boelelaan, Amsterdam, The Netherlands; 5 Reference Laboratory Neonatal Screening, Center for Health protection, National Institute for Public Health and the Environment, Bilthoven, The Netherlands; 6 Department of Laboratory Medicine, Laboratory Specialized Diagnostics & Research, Amsterdam UMC, University of Amsterdam, Meibergdreef, Amsterdam, The Netherlands; 7 Amsterdam Public Health, Amsterdam, The Netherlands; 8 Department of Endocrinology and Metabolism, Amsterdam UMC location University of Amsterdam, Meibergdreef, Amsterdam, The Netherlands; 9 TNO - Child Health, Sylviusweg, Leiden, The Netherlands; 10 Department of Paediatric Endocrinology, Emma Children’s Hospital, Amsterdam UMC, University of Amsterdam, Meibergdreef, Amsterdam, The Netherlands; 11 Amsterdam Reproduction & Development Research Institute, Amsterdam, The Netherlands; 12 Department of Pediatrics, Division of Metabolic Disorders, Emma Children’s Hospital, Amsterdam UMC, University of Amsterdam, Meibergdreef, Amsterdam, The Netherlands; 13 Department of Laboratory Medicine, Amsterdam UMC, Vrije Universiteit, Boelelaan, Amsterdam, The Netherlands; 14 Department of Laboratory Medicine, Amsterdam UMC, University of Amsterdam, Meibergdreef, Amsterdam, The Netherlands

**Keywords:** congenital hypothyroidism, newborn screening, amino acids, acylcarnitines, machine learning based

## Abstract

**Objective:**

Congenital hypothyroidism (CH) is an inborn thyroid hormone (TH) deficiency mostly caused by thyroidal (primary CH) or hypothalamic/pituitary (central CH) disturbances. Most CH newborn screening (NBS) programs are thyroid-stimulating-hormone (TSH) based, thereby only detecting primary CH. The Dutch NBS is based on measuring total thyroxine (T4) from dried blood spots, aiming to detect primary and central CH at the cost of more false-positive referrals (FPRs) (positive predictive value (PPV) of 21% in 2007–2017). An artificial PPV of 26% was yielded when using a machine learning-based model on the adjusted dataset described based on the Dutch CH NBS. Recently, amino acids (AAs) and acylcarnitines (ACs) have been shown to be associated with TH concentration. We therefore aimed to investigate whether AAs and ACs measured during NBS can contribute to better performance of the CH screening in the Netherlands by using a revised machine learning-based model.

**Methods:**

Dutch NBS data between 2007 and 2017 (CH screening results, AAs and ACs) from 1079 FPRs, 515 newborns with primary (431) and central CH (84) and data from 1842 healthy controls were used. A random forest model including these data was developed.

**Results:**

The random forest model with an artificial sensitivity of 100% yielded a PPV of 48% and AUROC of 0.99. Besides T4 and TSH, tyrosine, and succinylacetone were the main parameters contributing to the model’s performance.

**Conclusions:**

The PPV improved significantly (26–48%) by adding several AAs and ACs to our machine learning-based model, suggesting that adding these parameters benefits the current algorithm.

## Introduction

Congenital hypothyroidism (CH), an inborn thyroid hormone (TH) deficiency, can be divided into primary (or thyroidal) CH and central (or secondary) CH. Primary CH is most prevalent and results from an un(der)developed thyroid gland or a defect in TH synthesis. Central CH is caused by insufficient stimulation of an otherwise normal thyroid gland due to a defect at the level of the pituitary and/or hypothalamus. Primary CH is characterized by a low serum thyroxine (T4) concentration in combination with an increased thyroid-stimulating hormone (TSH) concentration, while central CH is characterized by a low T4 concentration and a normal, decreased, or slightly elevated TSH concentration (1).

The Dutch newborn screening (NBS) yearly screens approximately 170,000 newborns for, among others, CH. Blood withdrawal is normally performed between 72 and 168 h after birth and detects CH by measuring total T4 concentrations in every newborn as a first tier, expressed as standard deviation from the daily mean (T4_SD). In case of a T4_SD ≤ −0.8, TSH is measured (approximately the lowest 20% of the daily mean) and in case of a T4_SD ≤ −1.6, thyroxine binding globulin (TBG) is measured as well (approximately the lowest 5% of the daily T4 concentrations). TBG is used to reduce the number of false-positive referrals (FPRs) due to (partial) TBG deficiency which is associated with reduced total T4 concentrations in combination with normal free T4 (fT4) concentrations (2, 3). A calculated T4/TBG ratio serves as an indirect measure of fT4. A visual representation of this algorithm can be found in the review of Boelen *et al.* (4). If gestational age is ≤36 weeks or birthweight ≤2500 g, newborns are referred solely based on TSH concentration, since T4 concentration is not reliable yet. Most national NBS programs use TSH as primary marker, thereby not detecting central CH (5). In The Netherlands, the T4–TSH–TBG algorithm effectively detects primary CH as well as central CH, resulting in a higher prevalence (1:16,404) of central CH compared to other countries (6). A lower prevalence of primary CH was found, which may be explained by mild cases of primary CH with a normal T4 concentration that can be missed (7).

The downside of detecting primary and central CH is a low positive predictive value (PPV) of 21%, determined in the period of 2007–2017 (7). Our previous study aimed to improve the PPV of the Dutch CH screening algorithm by establishing reference intervals for T4 and TBG and thereby refining the cutoff value for a (partial) TBG deficiency (3) and by using machine learning (8). The latter study used a dataset containing almost all children with a referral in the historical CH screening from 2007 to 2017 (CH cases and FPRs) and children with normal CH NBS results from a cohort in 2019 (3). With machine learning (random forest model) using only general information and NBS CH screening results a PPV of 26% was obtained on this dataset, while all known CH cases were indicated as such by the algorithm. Thus, machine learning may have the potential to improve the PPV of the Dutch CH NBS. Nevertheless, the contrast with the PPV of a TSH-based screening is still large (67% in a UK cohort (9)). Besides TBG deficiency, another reason for FPRs can be non-thyroidal illness syndrome (NTIS), leading to a reduced T4 with a normal TSH concentration. To further improve the specificity of the Dutch NBS program for the detection of central CH, we sought for additional parameters. Recent studies describing an association between THs and serum amino acid (AA) and acylcarnitine (AC) concentrations (10, 11, 12, 13, 14) led us to explore the relationship between AAs, ACs, and the screening parameters T4, TSH, and the T4/TBG ratio in dried blood spots (DBS) at the time of neonatal screening. This is especially interesting since several AAs and ACs are measured in the NBS program to detect metabolic diseases. ACs play a role in the fatty acid β-oxidation under the influence of carnitine palmitoyltransferase 1 (CPT-1). The active TH triiodothyronine (T3) enhances the expression of the *CPT-1* gene, resulting in increased CPT-1 concentrations, thereby stimulating fatty acid β-oxidation (15). Recently, it has been shown that the AAs tyrosine (tyr) and its precursor phenylalanine (phe) correlate with serum T4 concentrations (12, 13). During TH synthesis, tyrosyl residues of thyroglobulin-forming iodotyrosines and iodothyronines are iodinated, which may explain the correlation with these AAs (16). Furthermore, it was shown that protein metabolism, shown by tyrosine and phenylalanine flux, was altered during the early stages of hyperthyroidism (17). The Dutch NBS program contains several metabolic disorders that are detected by a variety of AAs and ACs measured in DBS. Therefore, the aim of our study was to investigate whether the already known AA and AC concentrations could be of added value in the prediction of CH, and specifically central CH, in the Dutch NBS. We hypothesized that adding AA and AC concentrations to a CH prediction machine learning-based model could improve the PPV and thereby reduce the number of FPRs.

## Materials and methods

### Database

Parents participating in the Dutch neonatal screening program gave consent for both participation and anonymous use of leftover heel puncture blood and relevant data for scientific research, unless they actively declined. With the permission of the NBS privacy committee (located at The Netherlands Organization for Applied Scientific Research (i.e. TNO), Department of Child Health) and approved by the National Research Committee for Neonatal Screening (i.e. WONHS) of the Dutch National Institute for Public Health and the Environment (i.e. RIVM), data were extracted from a national database containing data of NBS referrals including both newborns with CH and FPRs in the period 2007–2017 (*n*= 3308) in succession of previously published studies aiming to further optimize the NBS for CH (7, 8). Permission from our local Medical Ethical Committee of Amsterdam UMC was not necessary, as the data were derived from a national database that is under the management of the abovementioned national institutes. The database contained the following variables: sex, gestational age, gestational weight, age at NBS samplings, T4, TSH, and TBG concentrations, T4_SD, T4/TBG ratio and the diagnosis. The T4/TBG ratio is calculated by the following formula: T4/TBG ratio = (T4_SD + 5.1) × 1000)/TBG. Due to the nature of the screening algorithm, TSH and TBG concentrations were not always available (*n* = 71 and *n* = 46) and subsequently imputed (see the section ‘Statistical analysis’). Four missing values were detected for T4.

Data of newborns with a normal CH screening result (hereafter named healthy controls) from a dataset from a recent study to establish neonatal reference intervals for T4, TSH, and TBG concentrations and the T4/TBG ratio were reused as healthy controls (3). This data (*n* = 1926) was collected between February and March 2019 at the Newborn Screening Laboratory of Amsterdam UMC, and for every subject, sex, gestational age, gestational weight, age at NBS sampling, T4, TSH, TBG concentrations, T4_SD, and the T4/TBG ratio were available. There were no missing T4, TSH, and TBG data.

Next, the already available AA and AC concentrations from both abovementioned cohorts were extracted from the Neonat Database of the Reference Laboratory for Neonatal Screening (RIVM) along with their year of birth, sex, gestational age, gestational weight, age at NBS sampling, and CH NBS results. The CH screening results and AC and AA data (C0, C2, C5, C5DC, C5OH, C6, C8, C10, C10:1, C14, C14:1, C14:2, C16, C16OH, C16:1, C18OH, phenylalanine, tyrosine, leucine, valine, succinylacetone) from the healthy control cohort were paired based on one unique parameter. Healthy controls were included if at least a T4, TSH, or TBG concentration and at least one AC or AA concentration were available and the coefficient of variation (CV) for T4, TBG, and TSH (manually assessed) was smaller than 20%. Furthermore, subjects with deviant AA or AC results suggestive of a metabolic disease were excluded. The CH screening results and AA and AC data from the referred cohort were paired using a manual multistep matching process, since no unique matching key was available. Subjects with identical values for a selection of variables were matched, and all one-to-one matches were extracted from the datasets. Matches that were not one-to-one (i.e. a subject in one dataset corresponds to multiple subjects in the other dataset) were investigated and manually adjusted. Finally, the data without a match were included in the next matching step. This was repeated five times for different sets of variables. The data that were not matched after these five steps were inspected manually in order to find additional matches that were not found before due to missing values or discrepancies. Referrals missing all TH parameters or all AA and AC data were excluded. Furthermore, referrals with an inconclusive diagnosis or CH with an unknown cause were excluded from the dataset as well as subjects with deviant AA or AC results suggestive of a metabolic disease. After matching, the datasets of referred newborns and nonreferred newborns were combined.

### Sample analysis

Heel prick punctures were performed between 72 and 168 h after birth and analyzed in one of the five regional laboratories in the Netherlands. The CH screening algorithm and the analyses of its parameters in our laboratory were extensively described (7). The measurement of AAs between 2007 and 2017 have been described previously (18) and the same method was used for the analysis of ACs. All five screening laboratories used the same measurement method (NeoBase 2 non-derivatized tandem mass spectrometry kit (PerkinElmer)) and the same cutoff values. All screening laboratories participated in external quality controls, and both intra- and interlaboratory variations were assessed and were within limits. Therefore, absolute values were comparable as well.

### Statistical analysis

First, the relationship between T4 and the AAs and ACs in all newborns was investigated to test our hypothesis that AA and AC concentrations were correlated to T4 concentrations. Pearson’s correlations were used, where the *P*-values were adjusted for multiple testing using the Benjamini–Hochberg procedure, reported as a false discovery rate (FDR) value. Data from newborns with a TBG concentration ≤105 nmol/L were excluded from the correlation analysis, as these subjects show a (partial) TBG deficiency and are currently not referred anymore since March 1, 2021 (3).

Second, linear regression analyses were performed to investigate whether AA and AC concentrations differed between the referred groups (referred controls, primary CH, central CH) and healthy controls. The Benjamini–Hochberg procedure was used to adjust the corresponding *P*-values for multiple testing. Regression analysis was not performed for ACs with less than ten unique values due to detection limit and sensitivity of the assay (C5, C5OH, C14:2, C16OH, C18:1OH).

Third, a random forest model was trained to predict the presence of CH. Prior to fitting the model, synthetic minority oversampling technique (SMOTE) (19) was used to create synthetic patient samples (*n* = 1612) based on the CH cases from the training set. The number of nearest neighbors for SMOTE was determined by evaluation of the performance for each integer between 5 and 50, and the best setting was 20 nearest neighbors. Randomized search cross-validation on the training set was used to select the values of four hyperparameters: the number of trees was set to 500, maximum depth of the trees to 15, five features were considered at each split, and 77% of the data were considered at each tree. The total dataset after matching with AA and AC data (*n* = 3436) was split in a training and test set (67%/33%) using a stratified split to preserve the prevalence of CH in both samples. Missing values were imputed using random forest-based imputation (20). The random forest was fit on the training set and thereafter used to impute the training and test sets. The F1 score was used as the performance metric for both hyperparameter tuning and model training. A receiver operating characteristic (ROC) analysis was performed to evaluate the performance of the model on the test set at different classification thresholds. PPV and accuracy of the model were calculated at a sensitivity set at 1.0, 0.99, and 0.98 (artificial sensitivity). The permutation feature importance was computed to investigate which parameters contributed most to the predictions (21). It is defined as the mean decrease in model performance in case one feature is randomly shuffled. The permutation feature importance was calculated on the whole test set as well as the test set without the primary CH cases, where the latter was used to determine which parameters contributed specifically to the prediction of central CH. The following variables were included in the model: sex, gestational age and weight, age at NBS samplings, T4, TSH, TBG concentrations, T4_SD, T4/TBG ratio, C0, C2, C5, C5DC, C5OH, C6, C8, C10, C10:1, C14, C14:1, C14:2, C16, C16OH, C16:1, C18OH, phenylalanine, tyrosine, leucine (leu), valine (val), succinylacetone (sa), and diagnosis.

## Results

### Sample characteristics

After applying exclusion criteria, the dataset consisted of 3436 newborns, of which 515 (15.0%) were diagnosed with CH, 1079 (31%) were FPRs, and 1842 (54%) were healthy controls. The percentages of primary CH and central CH in our database were 12.5% (431) and 2.4% (84), respectively.

### Relation between AA/AC and T4 levels (correlation analysis)

**Figure 1:**
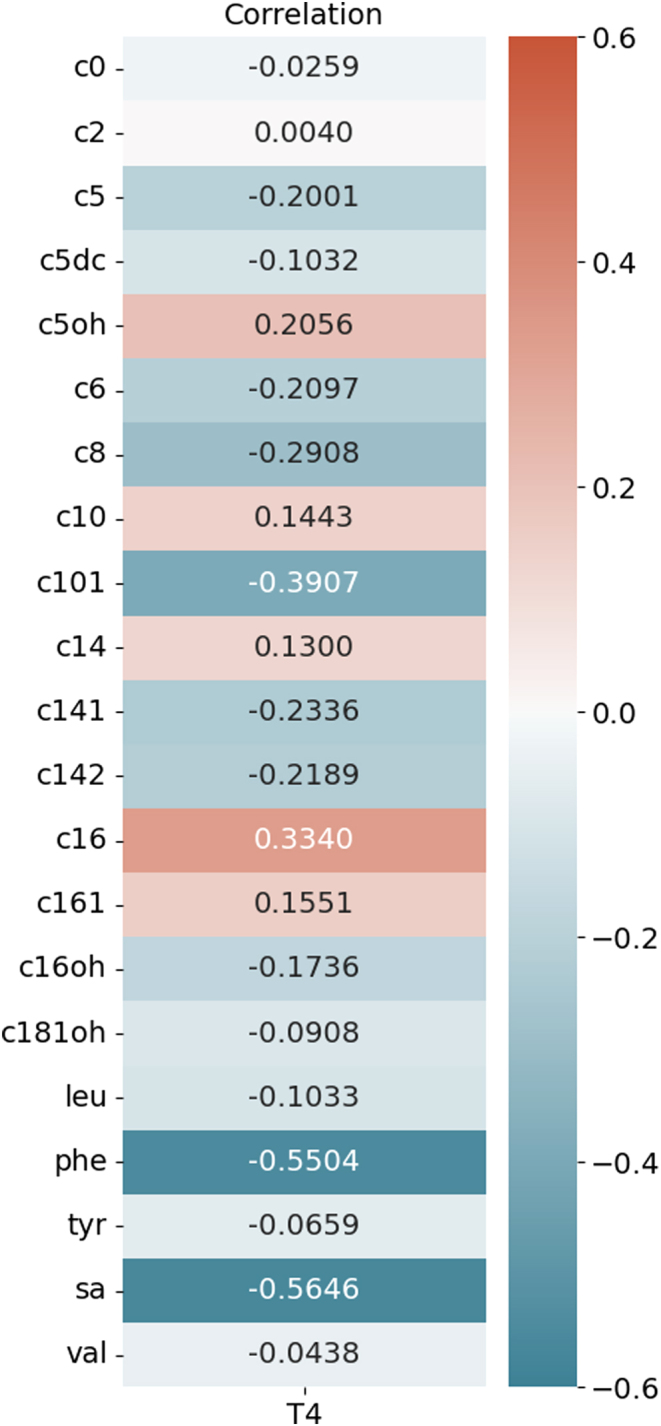
Pearson’s correlations (*ρ*) between the T4 concentrations and the AC and AA concentrations. All correlations were statistically significant (FDR < 0.05). AA, amino acid; AC, acylcarnitine; FDR, false discovery rate; leu, leucine; phe, phenylalanine; sa, succinylacetone; T4, thyroxine; tyr, tyrosine; val, valine.

The Pearson correlations between T4 and the measured ACs and AAs in all newborns are shown in Fig. 1. T4 concentrations correlated significantly (FDR < 0.05) with all ACs and AAs. However, the correlation coefficients (*ρ*) varied widely. Of the 16 ACs, T4 was most strongly correlated with C8 (*ρ* = −0.291), C10:1 (*ρ* = −0.391), and C16 (*ρ* = 0.334). The strongest correlations were between T4 and phenylalanine (*ρ* = −0.550) and succinylacetone (*ρ* = −0.565).

### Comparison of AC and AA levels between referred and non-referred cohorts (linear regression analyses)

Table 1 provides the results obtained from the linear regression analyses (after adjustment for multiple testing) to compare AC and AA results of the FPRs, primary CH and central CH cases with the healthy controls (HCs). The intercept represents the average concentration of the different ACs and AAs in the HCs, whereas the slopes are the additional value on top of the intercept that led to the concentrations of the different ACs and AAs in the FPRs, primary CH and central CH. Most ACs showed a statistically significant difference between HCs and FPRs, primary CH and central CH (Table 1). The difference between HCs and all other groups for phenylalanine and succinylacetone and the AC C10:1 showed the largest slopes and correlation coefficients (*R*^2^). Furthermore, a clear difference in tyrosine was found between HCs and central CH, whereas this was less evident between HCs and primary CH and non-existent between HCs and FPRs. In contrast, valine did not show any difference between HCs and central CH, whereas significantly lower concentrations were observed in primary CH compared to HCs and significantly higher concentrations in FPRs.

**Table 1:** Linear regression analysis including Benjamini–Hochberg adjustment for multiple testing to compare AC and AA results from the false-positive referrals (FPR), primary and central CH groups with the HC group.

	Intercept	FPR vs HC		Primary CH vs HC		Central CH vs HC		*R*^2^
		Slope	Adj. *P*	Slope	Adj. *P*	Slope	Adj. *P*	
AC								
C0	17.898	1.772	0.000	0.287	0.616	2.716	0.013	0.012
C2	15.451	0.947	0.007	1.289	0.008	3.283	0.002	0.007
C5DC	0.06	0.003	0.119	0.008	0.008	0.007	0.210	–0.001
C6	0.028	0.009	0.000	0.007	0.000	0.007	0.004	0.056
C8	0.032	0.016	0.000	0.009	0.000	0.009	0.002	0.093
C10	0.055	–0.004	0.000	–0.007	0.000	–0.001	0.731	0.015
C101	0.03	0.03	0.000	0.02	0.000	0.022	0.000	0.226
C14	0.158	–0.009	0.006	0	0.985	0.033	0.000	–0.003
C141	0.049	0.014	0.000	0.018	0.000	0.018	0.000	0.083
C16	2.705	–0.522	0.000	–0.559	0.000	–0.25	0.111	0.051
C161	0.145	–0.015	0.000	–0.008	0.135	–0.005	0.616	0.009
AA								
Leu	143.939	16.826	0.000	3.424	0.267	14.361	0.018	0.039
Phe	39.266	22.725	0.000	17.257	0.000	19.934	0.000	0.285
SA	0.133	0.282	0.000	0.265	0.000	0.262	0.000	0.429
Tyr	83.427	1.797	0.488	17.017	0.000	47.456	0.000	0.022
Val	134.185	7.69	0.000	–9.417	0.002	–0.451	0.952	0.027

AA, amino acids; AC, acylcarnitines; Adj., adjusted; Leu, leucine; Phe, phenylalanine; SA, succinylacetone; Tyr, tyrosine; Val, valine.

### Prediction model

**Figure 2:**
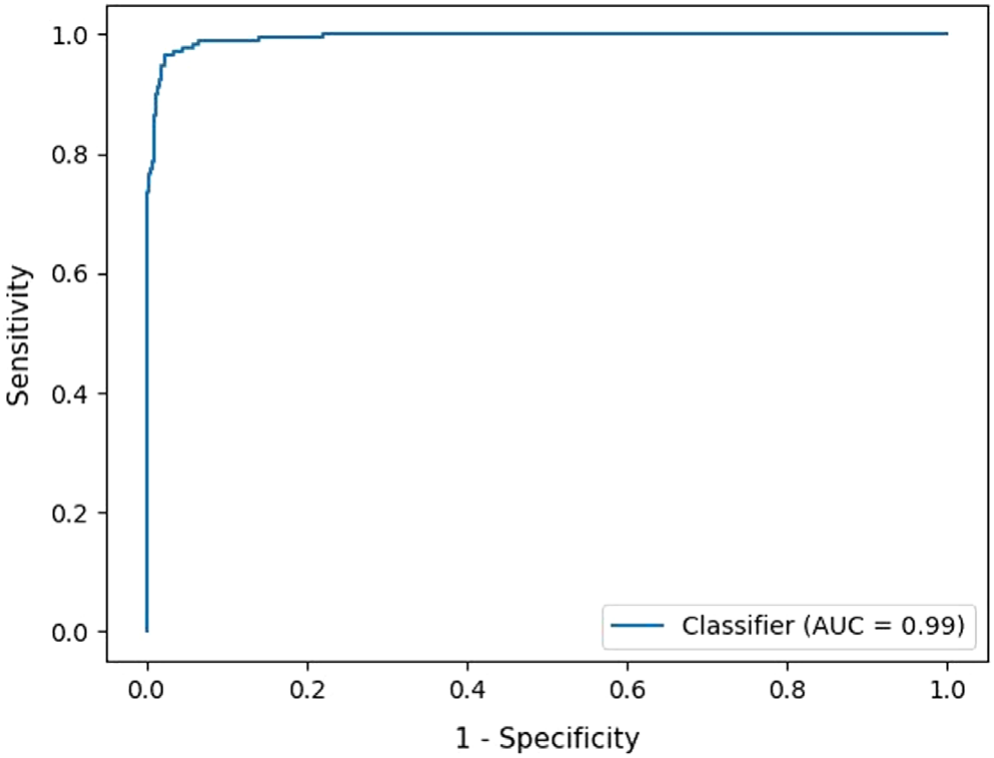
ROC analysis for the prediction of CH using a random forest model including AAs and ACs with an AUROC of 0.99. AAs, amino acids; ACs, acylcarnitines; AUROC, area under the ROC curve; ROC, receiver operating characteristic.

**Figure 3:**
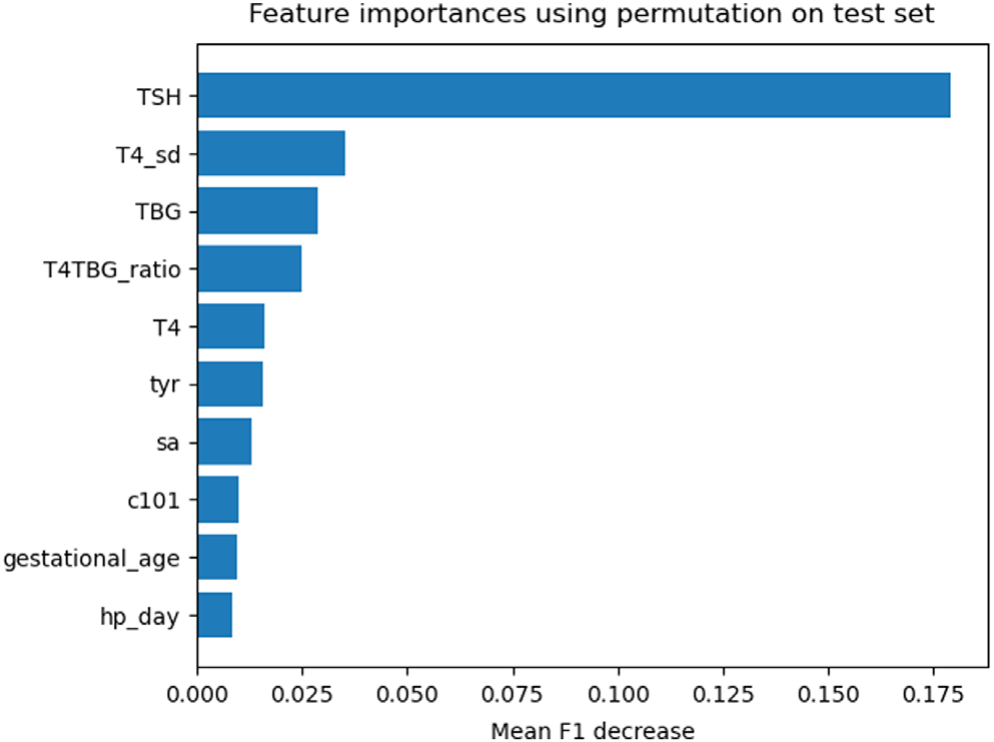
Permutation feature importance on the test set of the 10 parameters with the highest importance. hp_day, age at heel prick in days; phe, phenylalanine; sa, succinylacetone; T4_sd, thyroxine standard deviation; TBG, thyroxine-binding globulin;TSH, thyroid-stimulating hormone.

**Figure 4:**
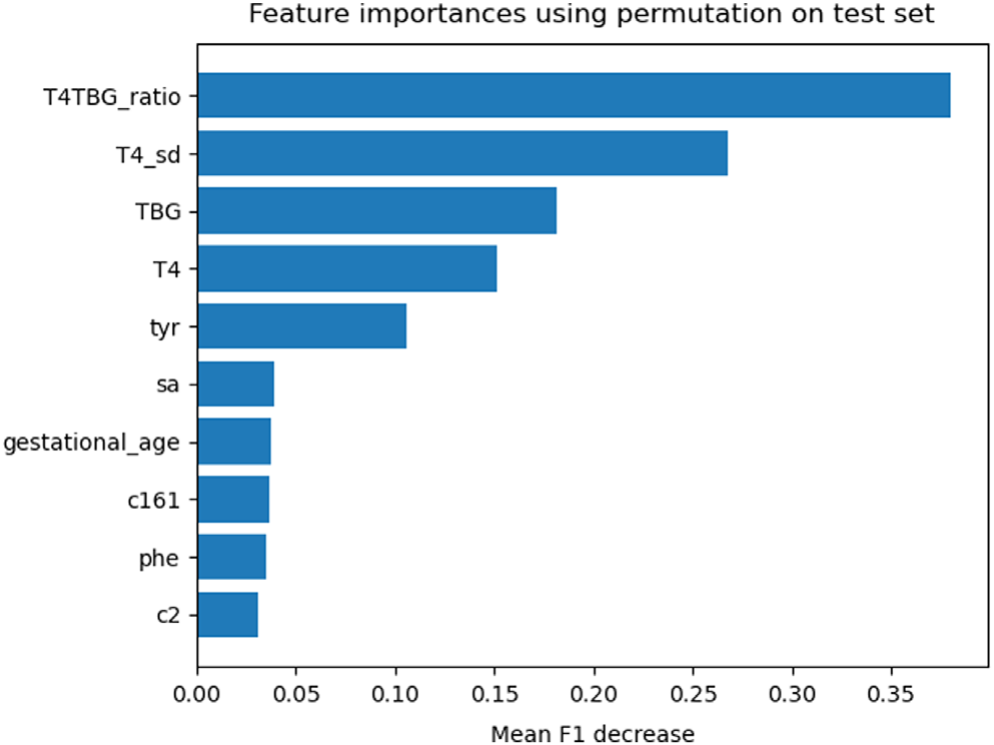
Permutation feature importance on the central CH cases, false-positive referred newborns and healthy controls in the test set of the 10 parameters with the highest importance. phe, phenylalanine; sa, succinylacetone; T4_sd, thyroxine standard deviation; TBG, thyroxine-binding globulin; tyr, tyrosine.

The PPV and accuracy of the random forest model, on the described database with relatively few healthy controls compared to the number of healthy controls in the actual 2007–2017 cohort, obtained for different sensitivities are shown in Table 2. The random forest model was able to yield an artificial sensitivity of 100%, while obtaining a PPV of 48%. Decreasing the sensitivity to 99% led to a substantial increase in PPV (65%). Figure 2 shows the ROC curve including the corresponding area under the ROC curve (AUROC) of 0.99. The permutation feature importance was estimated to quantify which parameters contributed most to the prediction of CH (Fig. 3). TSH had a mean F1 decrease around 0.175 and evidently played the most important role in predicting CH. Additionally, T4_SD, TBG, T4/TBG ratio, T4, and tyrosine contributed to the prediction of CH as well, followed by a lesser extent by succinylacetone, C10:1, gestational age, and age at NBS samplings in days (hd_day). Figure 4 shows which parameters were important in predicting central CH (Fig. 4). TSH has no explanatory value for these cases. The most important parameters for this model were the T4/TBG ratio, T4_SD, TBG, T4, and tyrosine followed by succinylacetone, gestational age, C16:1, phenylalanine, and C2.

**Table 2:** The positive predictive value (PPV) and accuracy reached by the model for different values of the artificial sensitivity (1.00, 0.99, 0.98), as well as the corresponding numbers of true-negative (TN), true-positive (TP), false-negative (FN), and false-positive (FP) results.

Sensitivity	PPV	Accuracy	TN	TP	FN	FP
1.00	0.48	0.84	781	170	0	183
0.99	0.65	0.92	874	168	2	90
0.98	0.82	0.96	927	166	4	37

## Discussion

The Dutch NBS program for CH is based on a T4–TSH–TBG algorithm aiming to detect newborns with either central CH or primary CH. Although the added value of the current screening program based on T4 is significant, this algorithm also results in more FPRs compared to TSH-based screening programs. An FPR often leads to stress and anxiousness in parents, which might even be transferred to the newborn (22). In an attempt to reduce the number of FPRs for CH, we investigated whether AAs and ACs could be of added value in the prediction of CH, and specifically central CH, in the Dutch NBS by using a machine learning-based model. Our study showed that the previously developed predictive machine learning-based model for the Dutch CH NBS can be optimized by adding AAs and ACs resulting in a strong improvement of the PPV of 26% in the study of Stroek *et al.* (8) up to 48% while preserving an artificial sensitivity of 100%. It should be mentioned here that our study population is not fully equal to that of Stroek *et al.* due to the availability or unavailability of AAs and ACs. These improvements are most likely caused by better discrimination between healthy controls and central CH. The large improvement of the PPV using this machine learning-based model does not yet equal the PPV of a TSH-based screening. However, the advantage of detecting primary and central CH instead of only primary CH is still considered most important. As the gap in PPV is now narrowing, this advantage even increases. A previous study using several NBS parameters and advanced modeling showed the possibility to reduce the amount of false-positively referred newborns (4–50%) in the CH NBS as well (23), both in TSH-based as T4-based screening programs. When specifically assessing their two T4–TSH based screening programs, reductions of false-positive cases with 17 and 31% were found. Similar to our approach, advanced modeling and additional markers that screen for unrelated conditions were used to improve the model. In contrast to our study, TBG was not assessed. Furthermore, a different approach was used (CLIR) and our study used all available AAs and ACs instead of a few selected ones (due to comparability with other centers in the study of Rowe *et al.*). In addition, the study by Rowe *et al.* already referred to a false-positive case if the first NBS indicated CH and the repeated NBS ruled out CH, whereas in the Netherlands a false-positive case is defined if a referred newborn is classified as healthy after a visit to the pediatrician. Although this study cannot be fully compared to our cohort due to the discrepancies mentioned here, it also highlighted the applicability of advanced modeling in improving the CH NBS by reducing the number of false-positive referrals using partially similar additional variables.

As expected, TSH played the most important role in our machine learning-based model in predicting CH. Since primary CH is the most common form of CH and characterized by an increased TSH concentration in the DBS, this subsequently will have the greatest impact on the model. T4, TBG, and the T4/TBG ratio are primarily used to detect central CH and were, therefore, also important. Interestingly, the AA tyrosine was a contributing parameter as well. When removing the primary CH group from the model to focus on the parameters that specifically predicted central CH, we confirmed that T4, TBG, T4/TBG ratio, tyrosine, succinylacetone, and gestational age were still important. Furthermore, C16:1, phenylalanine, and C2 contributed to the prediction of central CH. As expected, TSH did not contribute to the prediction of central CH at all. In accordance with the important predictive parameters, phenylalanine, succinylacetone, and C10:1 had the strongest correlation coefficients with T4. As a matter of fact, these parameters differed strongly between the groups in the linear regression analyses as well, thereby strengthening their added value. Tyrosine seemed especially powerful to distinguish central CH from HCs and FPRs explaining the importance of tyrosine as a parameter in our machine learning-based model.

TH parameters contribute most to the prediction of CH using this model, while the separate AAs and ACs contribute less. Indeed, THs and TSH are still key in diagnosing CH, but the addition of AAs and ACs may help to distinguish CH from HCs in some ‘difficult’ cases in the current algorithm. These difficult cases are most often FPRs that are mistakenly suspected of central CH. Although the individual contribution of AAs and ACs may be small, their cumulative effect on the prediction model is substantial as shown by the large improvement of the PPV by adding these parameters.

Previous research showed ambiguous results with both a positive and a negative association between fT4 and tyrosine, whereas the negative association of T4 with succinylacetone is new (10, 11, 12, 14, 24). The negative association between T4 and tyrosine might be explained by the fact that tyrosine is incorporated in the synthesis of THs (12, 16). However, it is not known if and how tyrosine might influence TH concentrations, so the exact nature of the relation is still unclear. As aromatic AAs, both tyrosine and phenylalanine seem to have an inhibitory effect on T3 uptake (25). Furthermore, it is noteworthy that tyrosine is particularly high in central CH. Central CH is mostly due to genetic mutations and is often associated with multiple pituitary hormone deficiency, an inborn deficiency of at least two anterior pituitary hormones (4). It is known that liver pathologies can increase tyrosine concentrations although the observed relation is difficult to explain (26, 27). Comorbidities to central CH might explain the selective increase in tyrosine in central CH. Succinylacetone is a by-product of the breakdown of tyrosine and relevant concentrations can only be detected when tyrosine metabolism is affected. This might explain why succinylacetone contributed as a parameter as well. However, we have to keep in mind that succinylacetone is a challenge to measure with the current method used for the metabolic screening, leading to less accurate results and more variation than desirable. Therefore, we cannot draw any definite conclusions regarding this biomarker yet. Once the measurement of succinylacetone becomes more accurate, the correlation with THs should be investigated again. The study of Chng *et al.* (12) found a positive association between tyrosine and fT4 concentrations in a small group of patients with hyperthyroidism before and after treatment. Our study, on the other hand, showed a negative association between those components (low T4 and high tyrosine). Further research is needed to clarify this discrepancy. Even though a connection between ACs and THs is present as discussed before, a causative relation is still lacking, and the mechanism involved is unknown. Several ACs contributed to the machine learning-based model to predict CH (C2, C10:1, C16:1); however, they played a smaller role compared to tyrosine and succinylacetone.

The strength of our study is that we included all primary and central CH cases from a large period of time in our dataset. We used a smaller healthy control cohort than the number of actual nonreferred newborns in the period 2007–2017. These healthy controls were necessary to get insight into results of parameters for nonreferrals. However, we acknowledge that this is not fully representative for the Dutch newborn screening population and results of our machine learning-based model can therefore not be 1:1 extrapolated to clinical practice. Nonetheless, this model attempted to mimic reality and its median T4, TSH, and TBG concentrations (described in the paper of Stroek *et al.* (3)) was proved to be comparable to the median T4, TSH, and TBG concentrations of the whole NBS population from 2008 to 2018 (7). Therefore, this model may be considered representative and could be reliably used. In March 2021, a new TBG cutoff value of 105 nmol/L was introduced to the screening. This has most likely improved the PPV of the NBS for CH already and adding AAs and ACs could improve the PPV even more. Therefore, a follow-up retrospective study cohort is necessary to validate our findings in a more recent dataset. Our machine learning-based model did not take false-negative newborns into account, as these cases are not well registered. Based on experience, approximately one to four false-negative results per year can be expected in the Dutch NBS program. Our machine learning-based model was built to have a sensitivity of 100% in our dataset, however this is an artificial sensitivity of 100% and thus cannot be compared to the Dutch screening population. Furthermore, tools should be provided to implement the predicted outcomes of a machine learning-based model in daily practice.

Another strength of our study is that we used AA and AC data from the NBS that were already measured for the screening of several metabolic conditions. Since these parameters are measured in every newborn, it can easily be implemented in our machine learning-based model to predict CH without much additional work or costs. Other AAs or ACs not yet measured may improve the model as well. However, the addition of the already available NBS data improved the model and did not lead to additional costs and is therefore preferred.

## Conclusion

In conclusion, we showed that the PPV of the previously developed predictive machine learning-based model for the Dutch NBS for CH was improved from 26% to 48% by adding several AAs and ACs. In particular, tyrosine contributed to this improvement. If this conclusion can be confirmed, implementation possibilities for this model need to be investigated, with the aim of reducing the number of false-positive referred newborns in the Dutch NBS for CH.

## Declaration of interest

There is no conflict of interest that could be perceived as prejudicing the impartiality of the research reported. A Boelen is on the editorial board of *European Thyroid Journal*; A Boelen was not involved in the review or editorial process for this paper, on which she is listed as an author.

## Funding

This research did not receive any specific grant from any funding agency in the public, commercial, or not-for-profit sector.

## Author contribution statement

HJ and MvH researched data for the article, made substantial contributions to the discussion of the content, wrote the article, and reviewed/edited the manuscript before submission. MB, WdE, EB, PvT, NZ-S, AH, AMB, and MH reviewed the manuscript before submission. CvdP and RdJ made substantial contributions to the content of the discussion and edited the manuscript before submission. AB designed the study, supervised the study, made substantial contributions to the discussion of the content, and reviewed/edited the manuscript before submission.
